# Government stewardship of the for-profit private health sector in Afghanistan

**DOI:** 10.1093/heapol/czw130

**Published:** 2016-09-28

**Authors:** Harry E. Cross, Omarzaman Sayedi, Laili Irani, Lauren C. Archer, Kathleen Sears, Suneeta Sharma

**Affiliations:** 1 Health Practice, Palladium, Washington DC; 2 Health Practice, Palladium, Kabul, Afghanistan; 3 The Population Reference Bureau, Washington DC

**Keywords:** stewardship, private health sector, governance, health expenditures, public–private partnerships, accountability and transparency

## Abstract

**Background**: Since 2003, Afghanistan's largely unregulated for-profit private health sector has grown at a rapid pace. In 2008, the Ministry of Public Health (MoPH) launched a long-term stewardship initiative to oversee and regulate private providers and align the sector with national health goals.

**Aim**: We examine the progress the MoPH has made towards more effective stewardship, consider the challenges and assess the early impacts on for-profit performance.

**Methods**: We reviewed publicly available documents, publications and the grey literature to analyse the development, adoption and implementation of strategies, policies and regulations. We carried out a series of key informant/participant interviews, organizational capacity assessments and analyses of hospital standards checklists. Using a literature review of health systems strengthening, we proposed an Afghan-specific definition of six key stewardship functions to assess progress towards MoPH stewardship objectives.

**Results**: The MoPH and its partners have achieved positive results in strengthening its private sector stewardship functions especially in generating actionable intelligence and establishing strategic policy directions, administrative structures and a legal and regulatory framework. Progress has also been made on improving accountability and transparency, building partnerships and applying minimum required standards to private hospitals. Procedural and operational issues still need resolution and the MoPH is establishing mechanisms for resolving them.

**Conclusions**: The MoPH stewardship initiative is notable for its achievements to date under challenging circumstances. Its success is due to the focus on developing a solid policy framework and building institutions and systems aimed at ensuring higher quality private services, and a rational long-term and sustainable role for the private sector. Although the MoPH stewardship initiative is still at an early stage, the evidence suggests that enhanced stewardship functions in the MoPH are leading to a more efficient and effective for-profit private sector. These successful early efforts offer high-leverage potential to rapidly scale up going forward.

Key messages

- Investing in Ministry of Public Health stewardship of the for-profit private sector can have a large impact by leveraging private sector resources to achieve broader health sector goals.
- The Ministry of Public Health has strengthened its stewardship function by improving the private sector policy and regulatory environment, establishing oversight structures and operating procedures and building human resource capacity.
- Applying minimum required standards to private hospitals is an important stewardship function for raising quality and accountability. Adapting minimum required standards to all private facilities promises to have a large positive impact on service quality.
- A committed government sharing a long-term common vision with its donors is essential to strengthening the stewardship role of the government in a low-income country like Afghanistan.

## Introduction

In the decade following the ousting of the Taliban in 2001–2003, the Afghanistan Ministry of Public Health (MoPH), with help mainly from USAID, the EU and World Bank, established a program to reconstruct and rapidly expand the country's basic health services. They contracted for a series of basic and essential package of health and hospital services (BPHS and EPHS) with non-governmental organizations (NGOs) and Provincial Health Offices while simultaneously building MoPH capacity to manage, monitor and evaluate the contracts (Newbrander *et al.* 2014). Because of this well-executed program, the country's basic health indicators improved significantly (Afghan Public Health Institute 2011). At the same time, Afghanistan's fragmented and largely unregulated for-profit private sector also grew at a rapid pace, accounting for nearly three-quarters of total health expenditures, but with little if any MoPH oversight.^1^ In many countries, the private health market is increasingly viewed as a critical component to expanding health services and achieving government health goals (Hort and Bloom 2013; Forsberg B *et al.* 2011; Lagomarsino *et al.* 2009). However, in Afghanistan, the MoPH has only recently begun to focus attention on the for-profit health sector as a contributor to the national policy of ′health for all′ (MoPH 2012b). Concerned by the large proportion of total health services provided by unregulated for-profit entities and resultant lack of quality, the MoPH began in 2008 to progressively adopt a combination of strategies, policies and regulations aimed at harnessing the for-profit health sector. The goal of these efforts was to help realize key government objectives of improving the quality of health care, achieving long-term sustainability and increasing private health investment. To carry out these strategic and policy mandates, the MoPH requested technical assistance from USAID, World Bank, EU and others, and launched an initiative to strengthen its stewardship capacity to oversee the for-profit private sector. In this article, we examine the progress the MoPH has made towards more effective stewardship, consider some of the challenges faced and assess the early impacts on for-profit performance.

While health stewardship is widely-accepted as a critical building block of health systems strengthening, how countries operationalize stewardship is a subject which has received little attention in the literature. With the adoption of the 2030 Sustainable Development Goals, in which the business sector is a key partner, countries' ability to effectively steward both the public and private sectors will be critical to achieving health targets—particularly universal health care and long-term sustainability (United Nations 2015). Nevertheless, health stewardship, especially of the for-profit private sector, is still weak in many countries. The Afghanistan case not only provides an analysis of how one country is building health stewardship capacity, but also offers lessons for other countries faced with similar needs to oversee and partner with the for-profit private sector.

## Role and characteristics of the for-profit private sector 

The overwhelming majority of health expenditures are household, out-of-pocket payments accounting for US$ 1.1 billion as shown in Table 1 for 2011–12. Donor financing, channelled to the public sector mainly through the MoPH, made up 21% of the total, while the Afghan government contributed just 5.6% of total health sector funding.

**Table 1.** Source of financing as a share of total Afghan health expenditures, 2011–2012

Source	Share
Public Sector	5.6%
Private sector*	73.6%
Donors**	20.8%
	100%

**Source:** MoPH 2013a. p. 13.

* All out-of-pocket except for 0.3% NGOs.

** Mainly, USAID, the EU, and World Bank.

Clients' visits by type of provider are presented in Table 2. Private providers account for almost half of all outpatient visits and more than one-in-six inpatient stays. The data also show that one-in-twenty Afghans sought inpatient or outpatient care in another county where they spent US$ 285 million, or more than a quarter of all out-of-pocket expenditures. The highest proportion spent abroad is indicative of the shortage of quality secondary and tertiary care in the country (MoPH 2013a).

**Table 2.** Use of Afghan health facilities by type of provider, 2011–2012 (%)

Type	Outpatient	Inpatient
Public hospitals/clinic*	53.0%	79.5%
Private hospital/clinic	45.1%	15.6%
Other**	0.4%	0.4%
Pharmacy	0.8%	–
Abroad	0.7%	4.5%
	100%	100%

**Source:** Central Statistics Organization 2014.

* Includes NGOs contracted for BHSP and EHSP services.

** Includes NGOs, nursing homes, mosques.

**Figure 1.**
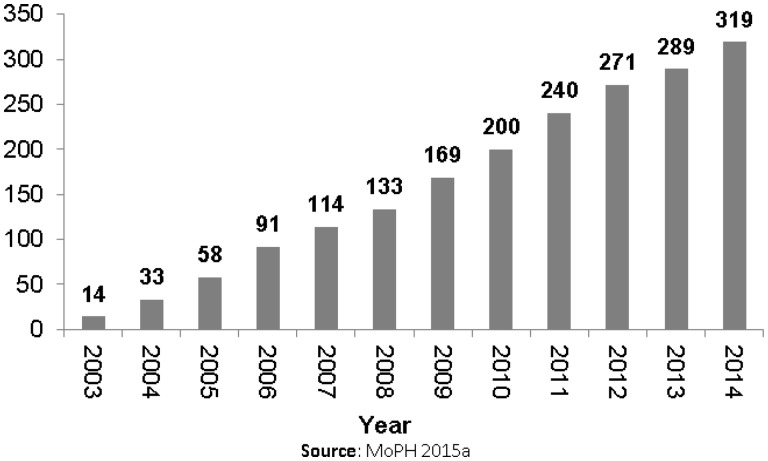
Cumulative number of private hospitals licensed, 2003–2014.

During the short period from 2008-09 to 2011-12, total health expenditures rose by 44% to over US$ 1.5 billion with the majority of new expenditures going to private providers (MoPH 2013a). Increased supply can be seen in Figure 1 which shows a five-fold rise in the cumulative number of private hospitals licensed by the MoPH in just 11 years (MoPH 2015a). Meanwhile, private laboratory and testing facilities and pharmacies grew 60 and 24%, respectively, between 2009/10 and 2013/14 (Central Statistical Organization 2009–14).

The rapid growth of the private sector also gave rise to concerns about the quality of for-profit health care. A 2008 private health sector survey revealed numerous deficiencies in training among Afghan private medical personnel. For example, in urban districts, one-in-five private providers had received no formal medical training. Poor infrastructure in terms of too few laboratory and diagnostic testing facilities, and inferior medicines also hindered quality. Client perceptions can often suggest quality concerns. Only 24 and 21% of private and public clients respectively rated quality of private services as ′positive′ with the remainder at ′adequate′ or ′negative′ (Global Health Technical Assistance Project. 2009). While the MoPH established mechanisms to assure minimal quality in the BPHS and EPHS packages, the for-profit private sector remained largely unregulated (Edwards *et al.* 2011).

## Health stewardship in the Afghan context

In 2000, the WHO concluded that health stewardship is the responsibility of ministries of health and it is the most important of the four vital functions of a health system (WHO 2000). Later, WHO codified stewardship as one of the six essential building blocks in its generally-accepted health systems framework (WHO 2007; WHO 2010). While there has been debate on the meaning and utility of the stewardship concept, there is concurrence on the core responsibilities of stewards as defined by WHO in 2009: ‘*ensuring that a strategic policy direction is formulated, ensuring good regulation and appropriate tools for implementing it and fostering the intelligence on health system performance needed to ensure accountability and transparency*’ (WHO 2009).

Until recently, stewardship of the for-profit health sector has not been a major focus of developing countries and Afghanistan was no exception (Lagomarsino *et al.* 2009; Akhtar 2011). Reasons for this were the government's entrenched negative perceptions of the for-profit private sector and a lack of private provider organizations to interface with the government. Additionally, there was an absence of comprehensive policies, structures and funding to enable the MoPH to exercise its stewardship responsibilities. As a result, the MoPH had little capacity and few mechanisms for regulating private providers. This began to change once the government and its donors came to appreciate the for-profit private sector’s key role and its potential contribution towards meeting MoPH health goals.

To define and analyse stewardship of the Afghan for-profit private sector, we propose six essential MoPH stewardship functions based on our review of the literature and our analysis of Afghan institutional developments. These six are closely aligned to the original stewardship functions found in WHO (2000) and Travis *et al.* (2002) later expanded upon by others (Veillard *et al.* 2011; Caulfield and Hort 2013; Alvarez-Rosete *et al.* 2013). In this article, they serve as a framework for analysing Afghanistan's progress towards establishing and exercising stewardship over the for-profit private health sector.

The essential stewardship functions are:

1. Formulating strategic policy direction
2. Ensuring the alignment of system structures with strategy and policy goals
3. Establishing the legal, regulatory and policy instruments to guide for-profit performance
4. Building and sustaining relationships, coalitions and partnerships
5. Ensuring accountability and transparency
6. Generating intelligence

## Methods 

To trace the development of the government’s emerging stewardship capacity and assess current status, we reviewed publicly available policy documents, publications and a substantial gray literature. We supplemented this research with a series of interviews and focus groups. Between December 2014 and March 2015, the third and fourth authors with the support of translators, carried out 31 semi-structured key informant interviews with various stakeholders including representatives of six MoPH departments/units involved in regulating the private health sector. We also surveyed key informants in the Ministry of Finance, Afghanistan Investment Support Agency and Afghan Chamber of Commerce and Industries. We interviewed representatives of private sector associations, including the Afghanistan Private Hospitals Association (APHA), Afghanistan Medicines Services Union (AMSU), the Afghan Midwives Association (AMA), Organization of Afghan Midwives (OAM) and Afghan Social Marketing Organization (ASMO). We surveyed nine hospital directors or owners who were members of APHA and ten owners of pharmaceutical companies/pharmacies/medical equipment suppliers who were members of AMSU. Additionally, we held three focus groups with 21 private providers, some of whom had undergone training offered by APHA on monitoring service quality, routine reporting of data to MoPH, infection prevention and waste management. Discussions with these individuals and groups focused on changes in the policy and regulatory environment, structural changes in MoPH and private sector stewardship, public–private communications and problem resolution, the implementation of the MoPH quality improvement guidelines for private hospitals (called *Minimum Required Standards* (MRS)), information collection and reporting, and prospects for strengthening public–private collaboration. All interviews were transcribed and translated into English. We used Atlas.ti, a qualitative analysis software, to code interviews and identify themes. Review of the publicly available documents and interviews were supplemented by analysis of data collected through routine inspections using the MRS checklist. To assess stewardship capacity, we conducted organizational capacity assessments of private associations including APHA, AMSU and AMA/OAM, and government units including the MoPH's Directorate of Private Sector Coordination (DPSC), Public–Private Partnership (PPP) Unit, and Health Economics and Financing Directorate (HEFD) during 2012–2015.

### Authors' Note

 This article describes and analyses more than 8 years of government and donor efforts to establish and improve MoPH stewardship over the for-profit health sector. This was a MoPH-led endeavour supported mainly by USAID, the World Bank and the EU involving hundreds of internal and external actors in differing capacities at different times. Various donor support programs provided technical and financial assistance to MoPH units, and private associations and groups. Four of the six authors worked directly on a USAID-funded project that provided periodic assistance to the DPSC and HEFD in the MoPH and to the APHA and AMSU in the private sector. All authors participated in carrying out the research activities as described in this section as a part of an overall assessment of MoPH private sector stewardship and routine project monitoring and evaluation.

## Results

In 2008, the MoPH began the lengthy process of building its stewardship capacity to oversee the for-profit private sector (Government of the Islamic Republic of Afghanistan [GIRoA] 2008). They also launched an effort to build the capacity of private providers to organize themselves, and with the MoPH, collaboratively guide private sector growth and performance. The theory of change was that establishing effective government stewardship of and collaboration with the for-profit health sector would expand health care to more Afghans, raise the quality of services and products, and in the long-term improve efficiency (MoPH 2009a). A greater uptake of private health services in urban areas would also theoretically free up future government and donor funds for targeting low-income rural populations. In short, by being an effective steward of the entire health sector and rationalizing the role of private providers, Afghanistan would improve its prospects for a stronger more sustainable health system.

### Formulating strategic policy direction

Formulating a strategic policy direction for the public and private sectors involves setting goals and objectives, and defining the government's responsibility and role including its stewardship functions. It also requires identifying the policy and institutional approaches as well as the sub-strategies and technical actions necessary for overseeing the private sector (Caulfield and Hort 2013; Travis *et al.* 2002; Veillard *et al.* 2011; Alvarez-Rosete *et al.* 2013). Table 3 summarizes the main strategies and implementation policies that describe the government's vision, rationale and technical directions for overseeing the for-profit private sector.

**Table 3.** Stewardship strategies and policies affecting the for- profit private health sectorz

Year	Document	Content/Noteworthy
2004	The Constitution of Afghanistan, 2004	Art. 52 calls for the expansion of the private health sector
2008	National Development Strategy, 2008–2013	Appoints and mandates strengthening the MoPH as the steward of the health sector
2009	National Policy for [the] Private Health Sector, 2009–2014 (MoPH)	Provides vision, guiding principles & policy directions for all types of for-profit health entities
2011	National Strategic Plan, 2011–15 (MoPH)	4 of 10 major strategies relate to growing and regulating the for-profit private sector
	Strategic Plan to Support Natl. Policy for Private Sector, 2009–14	Provides detailed plan of action to achieve objectives of the 2009 National Policy for [the] Private Health Sector
2012	National Health and Nutrition Policy, 2012–20 (revised 2015)	Revised policy underscores role of for-profit private sector and additional key areas for MoPH stewardship
2014	MoPH. 2014. Health Financing Strategy, 2014 – 2018	Has a special focus on insurance and public private partnerships
2015	National Policy for the Private Health Sector, 2015–20 (MoPH)	Strengthens 2009 policy for policy/reg. environment, public–private collaboration, MoPH stewardship (pending)

**Sources**: see References. Other supporting policies and strategies included: the National Strategy on Healthcare Financing and Sustainability (2009); Hospital Sector Strategy (2011); National Gender Strategy (2012); National Priority Program: Health for All (2012); National Reproductive Health Strategy, 2012-16 (2012); and the Afghanistan National Medicines Policy, 2014–2019 (2014).

Article 52 of the Afghan Constitution of 2004 promotes the for-profit private health sector by stating, ‘Establishment and expansion of private medical services as well as health centres shall be encouraged and protected by the state in accordance with…the law’. *The National Development Strategy*, 2008–2013, introduced the concept of health stewardship as a key component of the country's health development strategy stating that,  ‘ …the MoPH will work with … the private sector to coordinate the delivery of health care services by setting and distributing policies, standards and guidelines … ’ (GIRoA 2008). Within a year of the National Development Strategy, the MoPH followed up with a *National Policy for[the] Private Health Sector, 2009–2014*, which provided a vision and guiding principles for MoPH stewardship, a comprehensive list of policies needed for each type of business, and guidelines for developing the needed policies and regulations (MoPH 2009a).

The MoPH elevated the priority of for-profit stewardship in its *Strategic Plan, 2011–2015*, which set forth ten specific ‘strategic directions’ for the subsequent five years including ‘… *regulation and standardization of the private sector to provide quality health services*’ (MoPH 2011b). The plan committed the MoPH to developing the specific policies, regulations and procedures that would foster collaboration, communication and partnerships with the private sector. They also proposed enhancing the stewardship capacity of the MoPH and supporting evidence-based decision-making around the for-profit private sector (MoPH 2011e). The strategy was codified a year later in the *National Health and Nutrition Policy, 2012–2020* (MoPH 2012d).

### Ensuring the alignment of system structures with strategy and policy goals

This stewardship function ensures that policy goals are implemented through clear administrative structures, processes, procedures and the workforce necessary to effectively oversee private sector performance (Travis *et al.* 2002). Afghanistan's health sector strategies and policies originated at the highest levels of the MoPH, were actively supported mainly by USAID, World Bank and EU, and reflected a broad awareness of the importance of this stewardship function.

Between 2009 and 2015, the MoPH established new organizational structures and strengthened existing directorates and departments needed to implement the Ministry's private sector stewardship initiatives (see Table 4). The Office of Private Sector Coordination was established in 2009 to oversee most aspects of for-profit entities. Since then, it has helped implement policies, facilitate public–private engagement and communication, and advocate within the MoPH and across other ministries. In 2012, the Office created the PPP Unit to focus on designing, negotiating and managing large hospital PPPs, and encouraging health sector investment. Additionally, since 2010, the Office has implemented small-scale PPPs to support private hospital and clinic participation in national immunization and tuberculosis control programs (USAID 2013). While supported by donors and initially off-site, the Office of Private Sector Coordination was elevated to a Directorate (DPSC) in 2013 and officially integrated into the MoPH structure with a full complement of sanctioned civil service positions albeit, still largely supported by donors (Health Policy Project [HPP] 2015c).

**Table 4.** New health stewardship structures: for-profit private sector

Year	MoPH Unit	Function/event
2009	Office of Private Sector Co-ordination (OPSC)	Develops & implements policies & programs to engage the private sector
	Health Economics and Financing Directorate (HEFD)	Provides key data & analyses to policymakers and providers
2012	Public–Private Partnership Unit (sub-unit of DPSC)	Provides direction & oversight of MoPH PPPs
2013	Directorate of Private Sector Coordination (DPSC)	OPSC elevated to Directorate in MoPH
2015	Information & Communications Desk for Private Sector (DSPC)	One-stop door for licensing steps, approval forms, and regulations to obtain or renew a health business license

**Sources**: see References.

Recognizing the importance health economics and finance intelligence has for policy decision-making, the MoPH in 2009 established the HEFD which is responsible for collecting and analysing data, carrying out specialized studies, and fostering evidence-based policy dialogue and planning. HEFD subsequently carried out financial and policy analyses which were critical to understanding the for-profit private sector and thus strengthened MoPH policy analysis capacity (MoPH 2013d).

A long-standing structural issue led to the establishment of the Information and Communications Desk for Private Providers in 2015. The lengthy and fragmented process of licensing private general and specialty hospitals, clinics, radiology, diagnostic or physical therapy centres had long been a barrier to entry for prospective providers as well as current providers wishing to renew operating licenses. As one MoPH observer noted, ‘*Previously, the licensing process took too long. Even some investors stopped their activity and moved to other sectors*’. Thus, the DSPC and Licensing Department of the MoPH undertook an initiative to simplify and streamline the licensing process, reducing the number of steps required to obtain/renew a license from thirteen to six. Nonetheless, while the licensing procedures were simplified, the requirements to obtain a license and related regulations and policies were still difficult to access for private providers. To make the process more transparent and accessible, the DSPC organized the Information and Communications Desk as a ′one stop door′ to provide clients with all the information, forms and contact persons required to obtain or renew a license as well as the specific documents that are required for each of the licensing approval steps. The Desk also makes available all related policies and regulations related to licensing and operating a private health business.

Creating administrative structures was only part of establishing a stewardship function at the MoPH. Human resource capacity also needed to be built and strengthened. Government partners, mainly USAID, helped establish all the units in Table 4, including financing staff, equipment and activities and providing technical assistance in organizational and program development as well as staff training. Numerous other staff from the newly established units received on-the-job training through multi-year instructional programs, computer and software classes and one-on-one intensive training through joint project and research activities. As a result, these MoPH units were able to ramp up staff skills and experience to a point that they were viable and functioning as intended.

In sum, the MoPH has created a basic stewardship structure for overseeing the for-profit private sector with processes in place to regulate and collaborate with the private sector to obtain national health goals. An evaluation of DPSC between 2012 and 2014 found capacity gains in such areas as strategic planning, preparation of work and M&E plans, communicating technical updates, staff roles and responsibilities and staff satisfaction, but identified shortfalls including insufficient resources for M&E systems, lack of decisions informed by M&E data and inadequate records and information management (HPP 2015a). Assessments of the PPP Unit on the other hand, found that it had made substantial gains in stewardship and institutional capacity showing marked increases in the capacity of the indicators of highest importance and a corresponding reduction of indicators with less importance (HPP 2014b).

### Establishing the legal, regulatory and policy instruments to guide for-profit performance

Besides setting strategy and policy directions and establishing administrative structures, the MoPH required the means of operationalizing its stewardship responsibility. This meant concurrently developing a broad regulatory framework and policy instruments consisting of laws, regulations, decrees, standards and procedures to establish rules, incentives and sanctions for private providers. It also meant putting in place mechanisms for protecting the rights of consumers and providers. Table 5 shows the main for-profit-related regulations and operational policies and procedures adopted since 2012.

**Table 5.** Regulations, operational policies and procedures affecting the for- profit private health sector

Year	Action/Event	Purpose
2012	Private Health Centers Regulation	Regulations for establishing, licensing, paying fees, operating, monitoring private general & specialty hospitals, lab, radiology, other diagnostic facilities
	Public–Private Dialogue Forum	Quarterly meetings chaired by Minister of Health to solve problems & consult
2013	Minimum Required Standards for Private Hospitals (MoPH)	Technical guidelines for all aspects of private hospital operations w/focus on quality of services/care
2013	Health sector public–private partnership (PPP) regulations	Expands on procurement law w/respect to PPPs (under review)
2014	Decision Review and Sanction Committee (MoPH-in-process)	Redress and complaint mechanism for for-profit private sector; allows for review of sanction and other enforcement decisions
2015	National procurement law amended to include PPPs	Provides legal basis for undertaking PPPs in the health sector and across other sectors of economy
	MoPH ready to negotiate PPPs	All procedures for contracting out three major hospitals in place at PPP Unit

**Sources**: see References.

Passed by the Cabinet in 2012 after 5 years of discussion, debate and revisions, the *Private Health Centres Regulation* details the regulations, procedures and fees for establishing, licensing, and operating all private hospitals, clinics, and physical therapy and radiology centres. It has the force of law across the country and is the major legal means by which the MoPH exercises it stewardship authority over for-profit private sector providers (Forzley 2013; GIRoA 2012). Implementation of the *Regulation* rests with the MoPH which is charged with assigning responsibilities and developing necessary oversight mechanisms. The Legislation Implementation Ensuring Directorate has the main responsibility for discharging the *Regulation's* provisions and is supported by the M&E Department and the Food and Drug Quality Control Department.

Before its adoption, the proposed *Regulation* was modified by the Ministry of Justice whose staff were not familiar with private sector stewardship and market concepts. As a result, the law has several provisions that have caused concerns among current and prospective providers. For example, it calls for a MoPH commission to review and approve all fee schedules at private facilities. Other provisions propose potentially excessive monetary penalties for violators, high fees for licensing, overly stringent personnel requirements, and unnecessarily high capital guarantees (GIRoA 2012). Such stipulations could undermine enforcement, raise provider costs, discourage new entrants into the health market, and inhibit private provider expansion. Recognizing the potential adverse effect of certain provisions of the *Regulation*, the MoPH revised it in 2015 with more revisions in process. Successful implementation of the *Regulation* is dependent on the successful resolution of these potentially detrimental provisions.

A year after the *Regulation* was approved, the MoPH issued its *Minimum Required Standards*, which specify essential services, staffing, medical equipment and minimum physical configurations for hospitals. The MRS consists of 44 guideline categories covering all aspects of hospital, clinic and facility operations (MoPH 2013b). A scored checklist was adapted from the 44 MRS categories for use by private hospitals as a self-assessment mechanism for improving safety and service quality. The MRS was successfully tested with APHA hospitals and is being increasingly used by the MoPH as a monitoring tool for private providers. In this manner, the MoPH intends that the MRS will eventually help raise the quality of care among for-profit hospitals across the country.

Beginning in 2014, the APHA, representing for-profit hospitals, conducted collaborative MRS assessments with the MoPH of 41 of its 101 member hospitals. Of the total, 34% (14) scored above the 85% threshold for acceptable performance, and another 27% (11) were performing at the minimal level. Nearly 40% (16) of the assessed hospitals scored below the level required to maintain their licenses. Eight of the poor-performing private hospitals were selected for re-assessment six months after the first round. They showed average increases in performance of 8 percentage points (Sears *et al.* 2015).

During the interviews, hospital administrators and directors appreciated the benefits of universally-accepted standards and the MRS self-assessments. One hospital director clearly linked the MRS exercise to quality improvements: ‘*Once we implemented MRS, the mortality rate decreased and the treatment was more successful. The hospital has become standardized, the quality of care has gone up, and there is more infection prevention*’*.* Nevertheless, some APHA members expressed concerns about how the MoPH might apply provisions of the MRS. Lack of clarity about procedures caused some hospitals to worry about how the MRS can be implemented without corruption, adverse publicity and perceived lack of transparency in the processes. To address accountability and transparency in the stewardship system, the MoPH is in the process of establishing a Decision Review and Sanction Committee to provide private operators formal recourse for presenting evidence and contesting MoPH sanctioning decisions.

### Building and sustaining relationships, coalitions and partnerships

To be effective as a steward, a health ministry needs to build and sustain relationships, coalitions and partnerships inside and outside the government (Travis *et al.* 2002). The MoPH took a three pronged approach to this stewardship function by developing intergovernmental relationships, fostering public–private dialogue and promoting partnerships with private associations. The following illustrate these efforts.

#### Intragovernmental relationships

Within the Ministry, we have seen how the DPSC worked with the Licensing and M&E Departments and the Legislation Implementation Ensuring Directorate to simplify and streamline the private health centres licensing process. The PPP Unit worked closely with the Public Procurement Unit at the Ministry of Finance to amend the country's Procurement Law to include PPPs for health services (MoPH and HPP 2014). Similarly, the PPP Unit and allies collaborated with the Ministry of Finance and the Parliament to formally include health as a strategic sector in the Government's Investment Incentive Policy (GIRoA 2015).

#### Public–private dialogue

In the past decade, the MoPH has sponsored periodic working groups and task forces involving public, private and international participants, but these have mostly been called to work on specific strategies and policies. This changed in 2012 when the MoPH established a permanent *Public Private Dialogue Forum* chaired quarterly by the Minister. Attended by regulatory staff from MoPH departments and private sector representatives, the Forum deals with a range of legislative, regulatory and operational issues. The meetings have resulted in greater trust and collaboration between the MoPH and private providers, a consistent flow of information and the resolution of a number of regulatory issues affecting the private health sector.

#### Private sector partnerships

Effective MoPH stewardship required sufficient organizational and institutional capacity in the private sector to help providers begin to self-regulate, strengthen ethics and accountability, and interact with the MoPH on various regulatory matters. Fortunately, by 2012, several private associations were already functioning and could represent the private sector to the MoPH.

Established in 2007, the APHA has grown to include about one-third of all private hospitals in three major cities (Table 6). APHA representatives regularly participate in the *Public Private Dialogue Forum* and MoPH workshops. Most importantly, APHA represents member hospitals when there are regulatory problems or specific grievances. Another critical function of the APHA is to help member hospitals improve their quality of care. Thus, the association has sponsored in-service and clinical training especially to comply with *MRS* standards applicable to all private hospitals (Cross 2014).

**Table 6.** APHA Membership 2007–2014 (number of hospitals and city)

Year	Kabul	Herat	Mazar	Total
2007	10			
2008	13			
2009	36			
2010	36	10	5	51
2011	39	17	7	63
2012	41	20	7	68
2013	50	30	7	87
2014	56	33	12	101

**Source:** APHA 2014.

Formed in 2012, the 780-member strong AMSU is comprised of the country's main pharmaceutical and medical supplies and equipment associations and unions. One of its principal functions is to actively represent the interests of its members to the MoPH in the regulation of pharmaceutical quality and marketing. The AMSU has been an especially effective partner to the MoPH participating in 15 technical committees and working groups. It is an active member of the Public–Private Dialogue Forum, interacts regularly with the General Directorate for Pharmaceutical Affairs (GDPA), and sits on the National Medicines and Food Board. The AMSU routinely interacts with the MoPH to discuss and resolve regulatory and legal issues. As one member noted, ‘*If [the GDPA] cannot resolve an issue after two months, we are able to bring it up at the quarterly Dialogue Forums with the MoPH Minister*’*.*

### Ensuring accountability and transparency

The accountability function in a ministry of health commonly focuses on three broad areas of responsibility: financial, system performance and political accountability. The transparency function relates to how open and participatory a ministry exercises its main responsibilities internally and with the public (Brinkerhoff 2004, Kosack and Fung 2014).

The five other stewardship functions analysed in this paper all have elements designed to promote accountability and transparency. For example, the policies and strategies in Table 4 represent a multiyear collaboration among the government, private sector and international communities. As part of these processes, MoPH task forces and working groups were transparent and inclusive about the deliberations, design processes, preparation and review of strategies and policies. Once approved, the documents are posted on the MoPH website, but there is no formal mechanism yet for introducing and explaining policies and how the regulations will be administered. The DPSC has increased private sector participation in government committees, working groups and the Public–Private Dialogue Forum thus providing formal opportunities for MoPH and private sector representatives to transparently interact and problem-solve. The DPSC has also helped facilitate breakthroughs in accountability and transparency through the establishment of the forthcoming Decision Review and Sanction Committee and the Information & Communications Desk for health facility licensing (Sears *et al.* 2015).

The APHA and ASMU carried out activities and instituted procedures that strengthened their organizational governance as well as accountability and transparency between themselves and MoPH. With the MoPH, the APHA has sponsored various programs to help members be accountable in meeting government standards and more transparent in terms of services offered and posting prices. The AMSU has been working to improve accountability to the MoPH on drug quality. One importer observed in 2015 that, ‘*There is no way a company on its own can operate in a manner not approved by the organization…They know they are held accountable*’*.*

### Generating intelligence

There is general agreement in the literature that the collection, analysis and dissemination of data and information is essential for informed strategy, policy, regulatory and program decisions (Alvarez-Rosete *et al.* 2013). In Afghanistan, little was known about the private sector until a 2006 health survey showed high use of private providers for curative services even among the rural poor (Johns Hopkins University 2008). The first private sector health survey in 2008 also revealed heavy reliance on the for-profit private sector and spurred further interest in its potential by the MoPH (GHTA Project 2009). National Health Accounts (NHA) studies were undertaken in 2011 and 2013 that estimated private out-of-pocket payments by facility type and overall expenditures by sector (MoPH 2011d; MoPH 2013a). These NHAs were used to better understand the total health care market and the for-profit private sector. The 2013 NHA also quantified the enormous share of the country's health expenditure that was going overseas at the expense of the domestic market. This knowledge called further attention to the for-profit private sector and the importance of building MoPH stewardship capacity to help regulate and grow the sector. Two major surveys were in the field in 2015 and will shed further light on the role of the for-profit private sector (see Table 7).

**Table 7.** Key health data and analysis of the for-profit private sector

Year	Activity/Unit	Purpose
2006	Afghanistan Health Survey (AHS)	Revealed high level of private sector use even among poor.
2009	Private Sector Health Survey 2008 (USAID)	5-province survey to supplement AHS
2011	National Health Accounts 2008-09 (USAID)	Showed high levels of household expenditures go to private providers; helped inform policy discussions
2013	Capacity Assessment of the MoPH to Implement a New *Private Health Centers Regulation* (USAID)	Comprehensive legal and regulatory analysis of constraints and actions needed to implement the PHCR
2013	National Health Accounts 2011-2012 (HEFD)	Informs and guides MoPH private sector policy/strategies
2014	Health Management Information System for Private Hospitals (APHA)	Open source web based database for tracking 14 priority indicators and DEWS information
2015	A Health Insurance Feasibility Study in Afghanistan (HEFD)	Legal and stakeholder analysis, feasibility assessment highlights need for new health insurance law
	Afghanistan Demographic Health Survey (USAID)	National health survey, 25,600 household (expected 2016)
	Private Sector Health Assessment (World Bank)	National sample survey on private sector facility types, services, capacity, and quality. (expected 2016)

**Sources**: see References.

Of significance for MoPH intelligence generation is APHA's effort to develop a functioning Health Management Information System (HMIS) for private hospitals. A working group comprised of the MoPH, the APHA and other technical experts developed a set of indicators to be voluntarily reported to the APHA and subsequently to the Ministry. The MoPH M&E Department then signed an agreement with the APHA to roll out the private-hospital HMIS system. The system tracks 14 priority indicators as well as Disease Early Warning System information already sent to the MoPH by sentinel site private hospitals (MoPH 2013c). By the end of 2014, data from 60 hospitals had been reported at least once to the MoPH marking the first time it had access to private hospital health data (Sears *et al.* 2015).

## Discussion

Although significant progress has been made towards building public and private stewardship functions, there are important qualifiers that illustrate the challenges the MoPH and private sector partners face going forward. These are:

### 

#### Dependency on external funding and assistance

The MoPH’s and private associations’ stewardship-building activities were largely supported by external aid agencies–mainly USAID. This included subsidizing salaries, equipment and communications, training programs and operating costs as well as technical assistance and training. Although still supported by donors, the staff of the DPSC, PPP and HEFD offices are now ′on budget′ and integrated into the MoPH. The offices are still at risk if there are any cutbacks in assistance. In 2015, new support programs by the World Bank and USAID, aimed at institutionalizing and strengthening the new stewardship structures, extended assistance for another five years (World Bank 2013; USAID 2015). To succeed in the long term, MoPH stewardship programs will need to obtain government budget support and/or generate funding through fees. In the for-profit private sector, ASMU has made significant progress in its efforts to fund operations through membership fees and advertisements and is nearing financial self-sufficiency. While not as successful as ASMU, the APHA reports that 68 of its 100 hospitals are now paying full membership fees–up from <50 in 2014 (Chief Executive Officer, APHA, personal communication, November 5, 2015).

#### Policy implementation issues

Some aspects of regulations were inconsistent and possibly inhibited expansion of the for-profit private sector. Draft amendments to the *Private Health Centres Regulation* now are being considered that would remove key constraints such as the MoPH setting prices for private health centres and overly stringent human resource mandates. Key informants also noted that non-MoPH ministries have sometimes changed the way articles of the *Regulation* are applied without informing the MoPH or affected private health sector businesses. There is no mechanism yet in place to monitor the implementation of the Regulation so keeping track of policy implementation is a challenge.

#### Resistance to for-profit private health sector growth

Given Afghanistan's little prior experience with a mixed health system, it is not surprising that there are still pockets of resistance in the government to the concept of stewarding the private sector. However, concerns have declined substantially in the past few years as the benefits of incorporating the private sector into the MoPH's overall strategic plan became clearer. Examples such as PPPs around immunizations and family planning, emergency services, hospital referrals and the MRS are helping to promote support for such approaches (MoPH 2015c).

#### Minimum required standards implementation challenges

Just a few years ago, the MoPH had no standardized means of fairly assessing quality of care in private facilities, and no working relationships with private providers on regulatory matters. Progress on setting and applying mutually agreeable standards and opening channels of communication has been noteworthy. But private hospital operators remained concerned because of the absence of an appeal mechanism. The newly-approved *Decision Review and Sanction Committee* at the MoPH should alleviate some of these concerns. In 2015, the MoPH closed or put on probation a number of hospitals and reclassified others while offering all of them the opportunity to raise their standards against the MRS checklist (Adeel 2015). In taking these actions, the MoPH was exerting its stewardship of the private sector exactly in the manner it was intended to function. While these temporary closures may cause short-term issues for some private hospitals, it raises hopes for better quality in private hospitals going forward.

#### Health Management Information System

Private providers have also expressed some fears about the requirements to report service indicators to the MoPH. One hospital administrator noted, ‘* … people in the leadership in the hospitals have a concern that if they give the true numbers, there might be some consequences and backlashes from some entities like the Ministry of Finance. So far, there hasn’t been a complete understanding for what the reports are for and there is no mutual trust*’*.* Fear of releasing certain information was also reflected in statements suggesting that reporting requirements for APHA-member hospitals were unfairly applied. The private hospital reporting system is a critical component of the MoPH's stewardship program and the MoPH and its partners will do well to focus on increasing understanding of the utility of the HMIS in the next phase of assistance.

#### Large hospital PPPs delayed

After expedited legislative and capacity building efforts, the hoped-for PPP agreements between the MoPH and large investors for operating several donor-built hospitals have developed slowly. First, despite expressions of interest from international investors and World Bank loan and political risk guarantees, the MoPH has not garnered the necessary political commitment to issue formal bids for operating the hospitals. Second, prolonged presidential elections (2013–14), delays in appointing cabinet ministers and political uncertainty limited major government decision-making. In April 2015, the MoPH issued a public statement signaling its intention of entering into partnership with private investors to operate vacant tertiary hospitals in Kabul, and thus, the PPP initiative may move forward (MoPH 2015b).

#### Unfavourable security and economic conditions

In recent years, insecurity has increased both in the countryside and larger cities (Felbab-Brown 2015). Besides potentially inhibiting investors, the security situation delayed planned MoPH and partner activities that are part of the stewardship strengthening strategy. In addition, the economy has been harmed by the lack of clarity around future donor assistance and the uncertain political conditions during and after the 2014 elections. Achievement of the MoPH's stewardship goals will require adequate security and a continuance of favourable economic conditions.

#### Lessons learned

For many countries, achieving SDG health and equity goals by 2030 will not be possible without incorporating the for-profit private sector into overall government strategies to expand health services. Ministries of Health in LMIC countries therefore need to expand their stewardship capacity to align the private sector investment and services with SDG goals especially with respect to universal health care and long-term sustainability. In this context, there are important lessons to be learned from the Afghanistan experience described here.

The MoPH began to build and strengthen its health stewardship functions by putting in place comprehensive policies, strategies and codified approaches for overseeing the private sector. These policy and regulatory development processes required considerable time for deliberations, feedback and revisions. They were effective largely because they were carried out in a participatory and collaborative manner. Importantly, the effort would not have succeeded without the continuous political commitment by the government and its donors. Tracking and monitoring was essential for understanding the impacts of strategies, policy frameworks and regulations. A series of ′Policy Environment Score′ exercises conducted annually, policy analyses and periodic organizational assessments showed a variable but steady improvement in the for-profit private health sector policy environment. Not least, key stewardship initiatives were necessarily modest involving PPPs around preventive and reproductive care and applying minimum required standards to private hospitals to expand service availability, raise quality of care and improve accountability. These successful initial programs offer high-leverage potential to be rapidly scaled-up going forward.

## Conclusion

Investing in MoPH stewardship of the for-profit private sector has had a positive impact in Afghanistan by leveraging private sector resources to achieve broader health sector goals. Although the MoPH stewardship initiative is still at an early stage, the evidence suggests that with respect to the private sector, the health system in Afghanistan is functioning more effectively than it was five years earlier. The stewardship effort is promising precisely because, in contrast to the emergency years, the MoPH initiative has emphasized development of the institutional and regulatory systems of the MoPH aimed at ensuring the long-term sustainable growth of the private sector. However, progress to date must be seen as a solid first phase of a longer transformative process through which the results of the policies and strategies are fully sustained. Future progress stewarding the for-profit private sector, and the consequent expansion of a responsible and accountable private sector, will require a continuous, pro-active, coordinated leadership effort on the part of the MoPH to fully implement its stewardship functions.

As next steps, more research is needed to measure the impact of these approaches on improving health systems. Hence, we suggest a line of M&E that would yield important linkages between stewardship and health system outcomes going forward. In addition, this would help establish links to improved health system performance or health outcomes in the years to come (Hatt *et al.* 2015).

## Note

1 By for-profit private health sector, we mean the health care providers operating commercially as companies and individuals and under the regulatory oversight of the government.
